# Clinical Significance of B7‐H4 in Peripheral Blood of Patients With Myasthenia Gravis

**DOI:** 10.1155/jimr/2743928

**Published:** 2026-05-09

**Authors:** Xiaoling Zhou, Yunfei Zhu, Tiantian Gui, Yanzheng Gu, Haifeng Lu, Wentong Ju, Jingluan Tian, Qun Xue

**Affiliations:** ^1^ Department of Neurology, The First Affiliated Hospital of Soochow University, Suzhou, 215000, Jiangsu, China, sdfyy.cn; ^2^ Institute of Clinical Immunology, Jiangsu Key Laboratory of Clinical Immunology, The First Affiliated Hospital of Soochow University, Suzhou, 215006, Jiangsu, China, sdfyy.cn; ^3^ Department of Neurology, The Fourth Affiliated Hospital of Soochow University, Suzhou, 215123, Jiangsu, China; ^4^ Department of Neurology, The People’s Hospital of Guangxi Zhuang Autonomous Region, Guangxi Academy of Medical Sciences, Nanning, 530021, Guangxi, China, gxhospital.com

**Keywords:** autoreactive T cell, immunoregulation, membrane-bound B7-H4, myasthenia gravis, peripheral blood, soluble B7-H4

## Abstract

**Objectives:**

This study aimed to systematically investigate the dynamics changes in membrane‐bound B7‐H4 (mB7‐H4) expression and soluble B7‐H4 (sB7‐H4) levels in the peripheral blood of patients with acetylcholine receptor antibodies (AchR‐Ab)‐positive myasthenia gravis (MG) across different disease phases, and to explore their clinical significance.

**Methods:**

AchR‐Ab‐positive MG patients at baseline, relapse, and remission stages, along with age‐ and sex‐matched healthy controls (HCs), were enrolled. Peripheral blood was obtained from all participants. mB7‐H4 expression on circulating immune cell subsets was determined by flow cytometry, while plasma sB7‐H4 concentration was quantified using enzyme‐linked immunosorbent assay (ELISA).

**Results:**

Compared to HC, patients with relapsing MG exhibited significantly increased mB7‐H4 expression on CD4^+^ T cells and CD14^+^ monocytes (*p* < 0.05). In contrast, plasma sB7‐H4 levels were markedly decreased in relapsing‐MG patients relative to HC (*p* < 0.05). Among MG subgroups, mB7‐H4 expression on CD4^+^ T cells was significantly higher in relapsing‐MG patients than in those at baseline or in remission (*p* < 0.05). Conversely, plasma sB7‐H4 levels were significantly lower in both relapsing and remitting patients compared with the baseline‐MG group (*p* < 0.05). Within the relapsing‐MG patients, patients with abnormal thymic pathology exhibited significantly lower sB7‐H4 levels than those with a normal thymus (*p* < 0.05). Correlation analyses demonstrated that mB7‐H4 expression on CD14^+^ monocytes was positively correlated with quantitative MG scores (QMGs; *r* = 0.544, *p* = 0.020), whereas sB7‐H4 levels were negatively correlated with QMGs (*r* = −0.417, *p* = 0.016). Immunosuppressive treatment did not significantly affect sB7‐H4 levels compared with pre‐treatment values (*p* > 0.05). Univariate analysis identified elevated mB7‐H4 expression on CD4^+^ T cells as a potential risk factor for MG relapse. However, this association did not remain significant after multivariate adjustment for confounders variables.

**Conclusions:**

During MG relapse, inflammatory activation may engage the B7‐H4 pathway, characterized by increased mB7‐H4 and decreased sB7‐H4 levels, which may cooperatively contribute to immune suppression and serve as a compensatory protective mechanism. B7‐H4 expression appears to be associated with disease severity. Thymic abnormalities may contribute to the downregulation of sB7‐H4, whereas immunosuppressive treatment may not significantly modify its circulating levels. Although increased CD4^+^ B7‐H4 expression is associated with relapse, it does not represent an independent predictor of relapse risk.

## 1. Introduction

Myasthenia gravis (MG) is an autoimmune disorder characterized by pathogenic antibodies directed against acetylcholine receptors or functional‐related molecules located on the postsynaptic membrane at the neuromuscular junction. Clinically, MG manifests as fluctuating skeletal muscle weakness and fatigability [1]. Traditionally, MG has been regarded as a prototypical humoral immune‐mediated disease. With appropriate symptomatic, immunosuppressive, and supportive treatment, the majority of MG patients with mild‐to‐moderate symptoms can achieve complete remission or substantial clinical improvement [2]. However, complete remission remains uncommon in patients with refractory or severe MG cases following therapies that primarily target humoral immunity. This observation suggests that both humoral and cellular immune mechanisms may contribute critically to MG pathologenesis, underscoring the need to identify novel immunomodulatory targets.

T cells activation requires two distinct signals: antigen recognition through the major histocompatibility complex (MHC)‐peptide complex, and a second signal provided by costimulatory molecules. In the absence of adequate costimulatory signals, T cells fail to mount an effective immune response and may become anergic or undergo programmed cell death [3]. B7‐H4 (also named B7S1, B7x, or VTCN1) is an important coinhibitory molecule belonging to the B7/CD28 superfamily [4, 5]. Although B7‐H4 mRNA is widely expressed in normal tissues, its protein expression is tightly regulated, and the receptor of B7‐H4 is still unclear [6]. Functionally, B7‐H4 has been shown to promote tumor initiation and progression by facilitating immune evasion [7], while exerting protective effects in autoimmune settings such as type 1 diabetes and islet cell transplantation by suppressing both allogenic and autoreactive T‐cell responses [8–10]. Additionally, B7‐H4 plays important roles in organ protection, including the liver [11] and kidneys [12], as well as in the maintenance of pregnancy [13]. Notably, immunotherapeutic strategies targeting B7‐H4 (including monoclonal antibodies, antibody‐drug conjugates, anti‐B7‐H4/CD3 bispecific antibodies, and chimeric antigen receptor [CAR] T cells) have been successfully developed and have demonstrated promising therapeutic efficacy in preclinical and clinical studies [14–16].

To date, accumulating evidence has demonstrated that B7‐H4 is highly expressed in a variety of autoimmune diseases. In addition to its membrane‐bound form, B7‐H4 also exists in a soluble form (soluble B7‐H4 [sB7‐H4]) in plasma, which is frequently elevated under inflammatory conditions and in autoimmune disorders [17]. In rheumatoid arthritis (RA), Chen et al. [18] reported that B7‐H4 was expressed in synovial cells, CD34^+^ endothelial cells of neovessels, CD31^+^ endothelial cells, CD68^+^ macrophages, as well as in the membrane and cytoplasm of CD19^+^ B cells and CD14^+^ monocytes. Furthermore, B7‐H4 has been implicated in synovial remodeling and disease progression in RA [18]. Elevated serum sB7‐H4 levels in patients with RA are positively associated with disease activity, suggesting that sB7‐H4 may serve as a biomarker for disease diagnosing and monitoring [19]. In systemic lupus erythematosus (SLE), studies using murine models have shown that treatment with B7‐H4 antagonist antibodies exacerbates lupus manifestations, whereas administration of B7‐H4 immunoglobulin fusion proteins alleviated disease severity [20]. Consistently, clinical study have demonstrated that sB7‐H4 was overexpressed in patients with SLE, and its serum levels were negatively correlated with the SLE disease activity index score (SLEDAI) [21]. In type 1 diabetes, reduced B7‐H4 expression levels on pancreatic islet β‐cells is accompanied by diminished insulin expression [22]. Serum B7‐H4 levels were elevated in the early stages of disease progression and positively correlate with the disease progression [23]. Moreover, administration of sB7‐H4‐Ig fusion proteins effectively prevented the onset of diabetes in pre‐diabetic non‐obese diabetic (NOD) mice [8]. In neuroinflammatory disease models, Podojil et al. [24] demonstrated that B7‐H4‐Ig treatment significantly ameliorates disease progression in mice with relapsing and chronic experimental autoimmune encephalomyelitis (EAE). Conversely, depletion of B7‐H4 from C3H10 T1/2 mesenchymal stem cells can attenuate their immunomodulatory therapeutic effects in EAE model [25]. Collectively, these findings indicate that B7‐H4 represents a promising therapeutic target in autoimmune diseases. However, to date, no studies have systematically investigated the expression, function, or underlying mechanisms of B7‐H4 in MG and its role in MG pathogenesis remain unclear.

B7‐H4 is primarily expressed on activated T cells, B cells, monocytes, and dendritic cells (DCs) [6]. Accordingly, the present study aimed to investigate the expression patterns of membrane‐bound B7‐H4 (mB7‐H4) on CD4^+^ T cells, CD19^+^ B cells, and CD14^+^ monocytes in peripheral blood, as well as plasma levels of sB7‐H4, across different disease stages in patients with MG. We further analyzed the associations between B7‐H4 expression and clinical parameters and conducted preliminary explorations of the immunoregulatory role of B7‐H4 in MG. These findings are expected to elucidate the potential involvement of B7‐H4 in MG pathogenesis and provide a theoretical basis for the development of novel immune checkpoint‐based therapeutic strategies.

## 2. Methods

### 2.1. Patients and Controls

This study consecutively enrolled patients diagnosed with MG at the Department of Neurology, the First Affiliated Hospital of Soochow University, between July 2019 and June 2022. During the same period, race‐, gender‐, and age‐matched healthy controls (HCs) were recruited from the hospital’s physical examination center.

Inclusion criteria for MG patients were as follows: (1) diagnosis consistent with the Chinese Guidelines for the Diagnosis and Treatment of MG [26]; (2) adult patients aged 18 years or older, irrespective of sex; (3) classified into baseline, relapsing, or remitting MG subgroups; and (4) tested positive for acetylcholine receptor antibodies (AChR‐Ab).

Exclusion criteria comprised: (1) severe respiratory or circulatory system diseases; (2) presence of malignant tumors (with the exception of thymoma); (3) history of alcoholism, substance abuse, or psychiatric disorders; and (4) pregnancy or lactation.

Patients were categorized into the following subgroups:

Baseline‐MG: Patients at first onset who had not received glucocorticoids, immunosuppressants, plasma exchange, or intravenous immunoglobulin within the preceding 3 months.

Relapsing‐MG [27]: Patients meeting all of the following criteria: (1) had previously responded to treatment, reaching a status of minimal manifestations or better (per MGFA post‐intervention status); (2) experienced a recurrence of myasthenic symptoms lasting >24 h, occurring at least 30 days after the previous remission; and (3) showed an increase of ≥3 points in the quantitative MG score (QMGs) compared to the remission phase.

Remitting‐MG [28]: Patients with no detectable myasthenic signs or symptoms upon careful examination, allowing for possible isolated eyelid closure weakness.

AchR‐Ab positivity was defined as a plasma concentration ≥0.45 nmol/L, as measured by a commercial enzyme‐linked immunosorbent assay (ELISA) kit (RSR Ltd., UK).

### 2.2. Standard Protocol Approvals and Patient Consents

The study protocol was approved by the Ethics Committee of the First Affiliated Hospital of Soochow University. Written informed consent was obtained from all participants prior to study enrollment.

### 2.3. Clinical Data

Demographic and clinical parameters were collected from the MG patients, including sex, age at onset, MGFA classification, disease duration, AchR‐Ab concentration, thymus histopathology, treatment regimens, and QMGs. Disease duration was calculated as the interval from the first onset of muscle weakness to the time of blood collection at study enrollment. Disease severity was assessed using the QMGs.

### 2.4. Sample Processing

Fasting venous blood was collected in the morning from all participants in two EDTA‐ anticoagulated tubes. One tube was utilized for immunofluorescence staining and flow cytometry analysis. Another tube was centrifuged at 3000 rpm for 20 min, after which plasma was harvested and stored at approximately −80°C until further analysis.

### 2.5. Immunofluorescence Labeling and Flow Cytometry Analysis

#### 2.5.1. Collection and Staining

Fifty microliters of peripheral blood was collected from each participant and incubated with PE‐conjugated anti‐B7‐H4, PC5‐conjugated anti‐CD4, FITC‐conjugated anti‐CD19, and FITC‐conjugated anti‐CD14 antibodies (BioLegend) for 30 min at room temperature in the dark.

#### 2.5.2. Erythrocyte Lysis

Red blood cells were lysed by adding 200 μL of erythrocyte lysis buffer (Beckman Coulter) and incubating for 10 min at 37°C.

#### 2.5.3. Wash and Resuspension

The samples were washed with 1 mL of PBS, centrifuged at 2000 rpm for 5 min, and resuspended in 500 μL of PBS.

#### 2.5.4. Acquisition and Analysis

Sample acquisition was performed on a flow cytometer (Beckman Coulter), and the resulting data were analyzed using FlowJo software (version 10.4).

### 2.6. ELISAs

#### 2.6.1. Sample Preparation

Frozen plasma samples were thawed at room temperature and centrifuged at 2000 rpm for 5 min to obtain a clear supernatant.

#### 2.6.2. ELISA Procedure

Plasma sB7‐H4 concentrations were measured using a human sB7‐H4 ELISA kit (Shanghai Kang Lang Biological Technology Co., Ltd.) according to the manufacturer’s instructions. Each sample was diluted 1:5, and 50 µL of the dilution was loaded into the wells in duplicate.

#### 2.6.3. Quantification

The optical density (OD) was measured at 450 nm using a microplate reader. A standard curve was generated, and the concentration of sB7‐H4 in each sample was calculated and expressed as pg/mL.

#### 2.6.4. Quality Control

Negative and positive controls were included in each assay to ensure assay reliability.

### 2.7. Statistical Analyses

All statistical analyses were performed using SPSS (version 26.0) and GraphPad Prism (version 8.0) software. Continuous variables are presented as mean ± standard deviation (if normally distributed) or median with interquartile range (if non‐normally distributed). Comparisons between two independent groups were made using the Student’s *t*‐test (normal distribution) or the Mann–Whitney *U* test (non‐normal distribution). For comparisons across more than two groups, one‐way ANOVA (normal distribution) or the Kruskal–Wallis test (non‐normal distribution) was applied, with post hoc pairwise comparisons corrected using the Bonferroni method. Categorical data are expressed as frequencies and percentages and were compared using the Chi‐square test or Fisher’s exact test, as appropriate. Correlations between continuous variables were assessed with Pearson’s (normal distribution) or Spearman’s (non‐normal distribution) correlation coefficient. To identify potential risk factors for MG relapse, univariate logistic regression was first performed. Variables with a significance level of *p* < 0.05 in the univariate analysis were subsequently included in a multivariate logistic regression model using the enter method. The results are presented as odds ratios (ORs) with 95% confidence intervals (CIs). All tests were two‐sided, and a *p*‐value of less than 0.05 was considered statistically significant.

## 3. Results

### 3.1. Subject Characteristics

To investigate the membrane‐bound (mB7‐H4) and soluble (sB7‐H4) forms of B7‐H4, the study population was divided into two independent cohorts. As summarized in Table 1, the mB7‐H4 cohort comprised 16 baseline‐MG, 18 relapsing‐MG, and 29 remitting‐MG patients, along with 30 HC. The sB7‐H4 cohort comprised 31 baseline‐MG, 33 relapsing‐MG, 29 remitting‐MG patients, as well as 30 HC. No significant differences were observed among groups with respect to sex or age (all *p* > 0.05).

**Table 1:** Demographic and clinical characteristics of study participants.

Characteristics	mB7‐H4					sB7‐H4				
	Baseline	Relapsing	Remitting	HC	*p*	Baseline	Relapsing	Remitting	HC	*p*
No. of cases	16	18	29	30	—	31	33	29	30	—
Age (years)	45.63 ± 12.60	48.39 ± 17.23	48.79 ± 17.44	47.03 ± 18.19	0.933	47.13 ± 18.55	49.61 ± 13.46	45.03 ± 17.04	40.67 ± 14.88	0.054
Gender, *n* (%)	—	—	—	—	0.534	—	—	—	—	0.108
Female	7 (43.7)	12 (66.7)	14 (48.3)	15 (50.0)	—	15 (48.4)	19 (57.6)	20 (69.0)	23 (76.7)	—
Male	9 (56.3)	6 (33.3)	15 (51.7)	15 (50.0)	—	16 (51.6)	14 (42.4)	9 (31.0)	7 (23.3)	—
Disease duration (months)	4.00 (1.00, 24.00)	24.00 (12.00, 41.00)	21.00 (8.00, 59.00)	NA	0.036	1.00 (1.00, 2.00)	20.00 (3.00, 52.00)	15.00 (10.00, 38.00)	NA	0.000
MGFA classification, *n* (%)	—	—	—	—	0.001	—	—	—	—	0.000
OMG	10 (62.5)	3 (16.7)	21 (72.4)	NA	—	20 (64.5)	2 (6.1)	18 (62.1)	NA	—
GMG	6 (37.5)	15 (83.3)	8 (27.6)	NA	—	11 (35.5)	31 (93.9)	11 (37.9)	NA	—
Thymus pathology, *n* (%)	—	—	—	—	0.003	—	—	—	—	0.026
Normal thymus	16 (100)	9 (50.0)	23 (79.3)	NA	—	21 (67.7)	13 (39.4)	20 (69.0)	NA	—
Abnormal thymus	0 (0)	9 (50.0)	6 (20.7)	NA	—	10 (32.3)	20 (60.6)	9 (31.0)	NA	—
AchR‐Ab Concentration (nmol/L)	6.92 ± 4.99	11.36 ± 3.40	7.86 ± 4.39	NA	0.007	9.01 ± 4.80	11.49 ± 3.80	6.67 ± 4.10	NA	0.000
QMGS	6.00 (5.00, 11.50)	13.50 (11.00, 18.00)	7.00 (6.00, 10.00)	NA	0.000	8.00 (6.00, 12.00)	14.00 (12.00, 20.00)	7.00 (6.00, 9.00)	NA	0.000
Treatment, *n* (%)										
Pyridostigmine	11 (68.8)	16 (88.9)	19 (65.5)	NA	0.194	26 (83.9)	30 (90.9)	16 (55.2)	NA	0.002
Immunotherapy										
Glucocorticoid	NA	16 (88.9)	21 (72.4)	NA	0.000	NA	32 (96.7)	21 (72.4)	NA	0.006
Azathioprine	NA	1 (5.6)	6 (20.7)	NA	0.085	NA	3 (9.1)	4 (13.8)	NA	0.559
Tacrolimus	NA	3 (16.7)	10 (34.5)	NA	0.184	NA	1 (3.0)	9 (31.0)	NA	0.003
MMF	NA	1 (5.6)	1 (3.5)	NA	0.843	NA	1 (3.0)	0 (0.0)	NA	0.345
RTX	NA	0 (0.0)	2 (6.7)	NA	0.255	NA	0 (0.0)	0 (0.0)	NA	NA
CTX	NA	1 (5.6)	1 (3.5)	NA	0.728	NA	2 (6.1)	2 (6.9)	NA	0.894

Abbreviations: AchR‐Ab, acetylcholine receptor antibody; CTX, cyclophosphamide; GMG, generalized myasthenia gravis; HC, healthy control; mB7‐H4, membrane B7‐H4; MG, myasthenia gravis; MGFA, myasthenia gravis foundation of America; MMF, mycophenolate mofetil; NA, not applicable; OMG, ocular myasthenia gravis; QMGs, quantitative myasthenia gravis score; RTX, rituximab; sB7‐H4, soluble B7‐H4.

### 3.2. B7‐H4 Expression Across Disease Stages

A representative gating strategy for B7‐H4 expression in CD4^+^ T cells, CD19^+^ B cells, and CD14^+^ monocytes from MG patients and HCs is shown in Figure 1A,B. Detailed sequential gates are provided in Figure S1. As illustrated in Figure 2A, mB7‐H4 expression on CD4^+^ T cells was significantly upregulated in relapsing‐MG patients compared to HC (*p* < 0.05). In contrast, no significant differences were observed between the baseline‐MG or remitting‐MG groups and HC (*p* > 0.05). Furthermore, mB7‐H4 expression on CD4^+^ T cells was significantly higher in the relapsing‐MG group than in both the baseline‐MG and remitting‐MG groups (*p* < 0.05), whereas no difference was detected between the latter two groups (*p* > 0.05).

Figure 1Representative gating strategy and comparison of B7‐H4 expression levels in peripheral blood immune cells from myasthenia gravis (MG) patients and healthy controls (HC). (A, B) Representative flow cytometry gating strategy for B7‐H4 in CD4^+^ T cells, CD19^+^ B cells, and CD14^+^ monocytes from the MG (A) and HC (B) groups. Representative histograms for the indicated subsets are shown stained with a fluorescence minus one (FMO) control (blue) or B7‐H4 (red). (C–E) Summary data comparing B7‐H4 expression levels between MG (*n* = 63) and HC (*n* = 30) groups on (C) CD4^+^ T cells, (D) CD19^+^ B cells, and (E) CD14^+^ monocytes. Data are presented as mean ± standard deviation (SD). Statistical analysis was performed using unpaired Student’s *t* test.  ^∗∗^ *p* < 0.01; ns, not significant.

**Figure:**
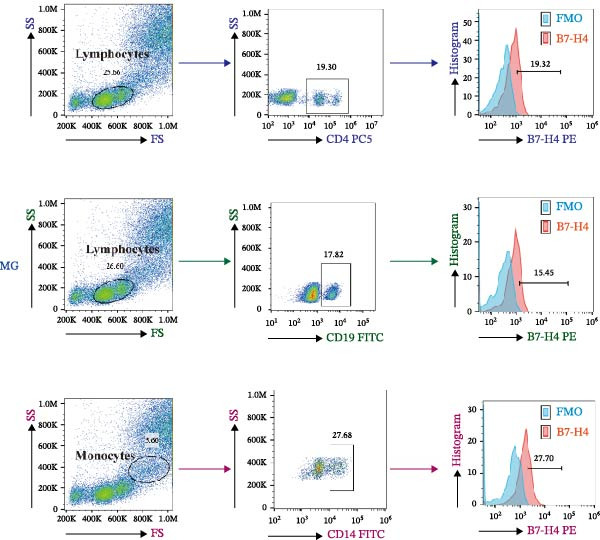
(A)

**Figure:**
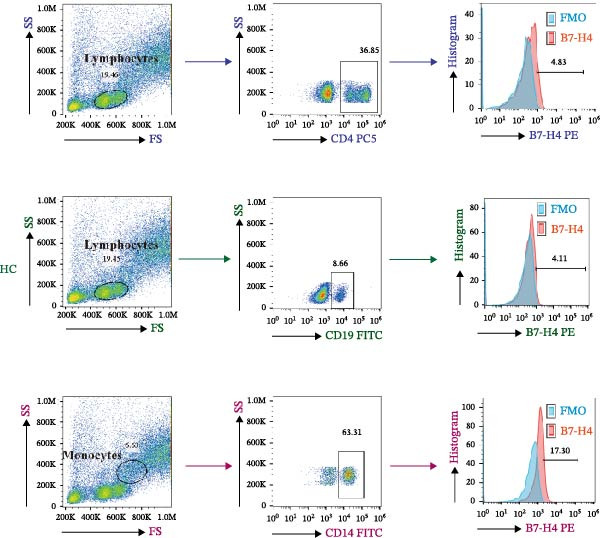
(B)

**Figure:**
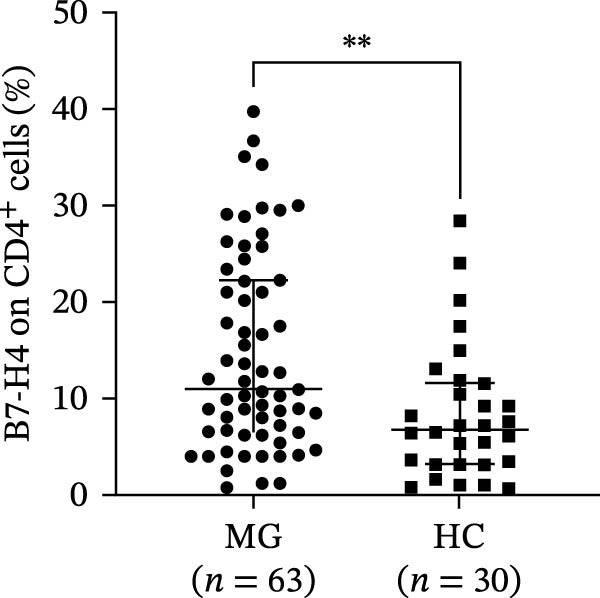
(C)

**Figure:**
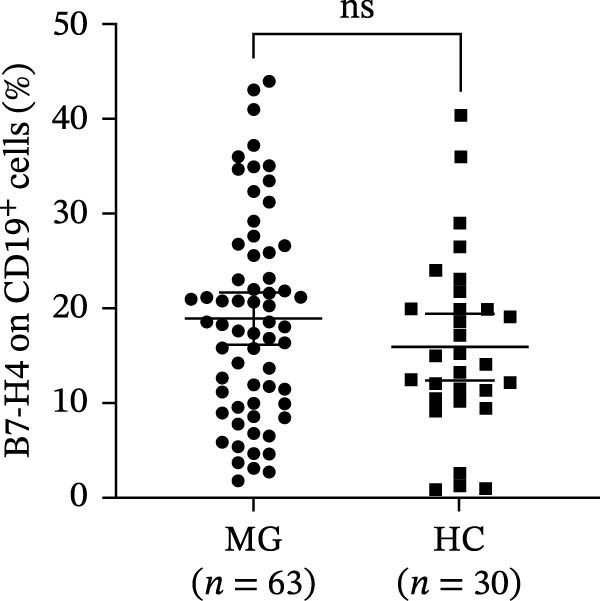
(D)

**Figure:**
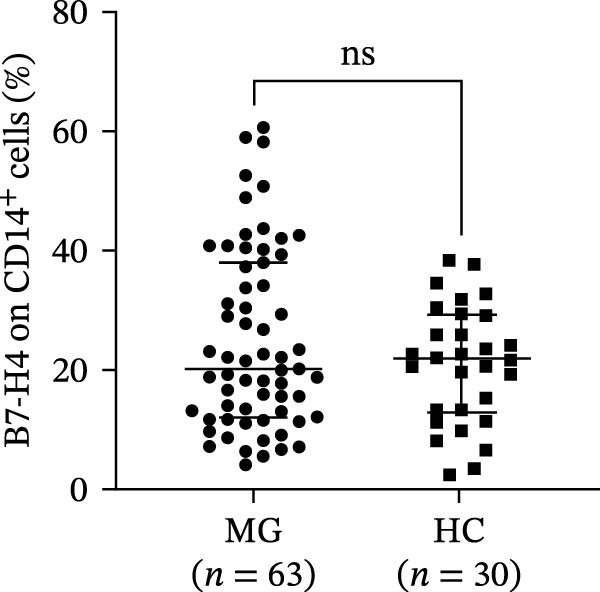
(E)

Figure 2Expression levels of membrane‐bound B7‐H4 (mB7‐H4) on peripheral blood immune cells and soluble B7‐H4 (sB7‐H4) in plasma across different disease stages of myasthenia gravis (MG) and in healthy controls (HC). (A–C) Percentage of B7‐H4^+^ cells within (A) CD4^+^ T cells, (B) CD19^+^ B cells, and (C) CD14^+^ monocytes from baseline‐MG (*n* = 16), relapsing‐MG (*n* = 18), Remitting‐MG (*n* = 29), and healthy controls (*n* = 30). (D) Plasma soluble B7‐H4 (sB7‐H4) concentrations in baseline‐MG (*n* = 31), relapsing‐MG (*n* = 33), remitting‐MG (*n* = 29), and HC (*n* = 30). Data are presented as mean ± standard deviation. Statistical analysis was performed using one‐way ANOVA, with post hoc pairwise comparisons corrected using the Bonferroni method.  ^∗^ *p* < 0.05,  ^∗∗^ *p* < 0.01,  ^∗∗∗^ *p* < 0.001,  ^∗∗∗∗^ *p* < 0.0001; ns, not significant.

**Figure:**
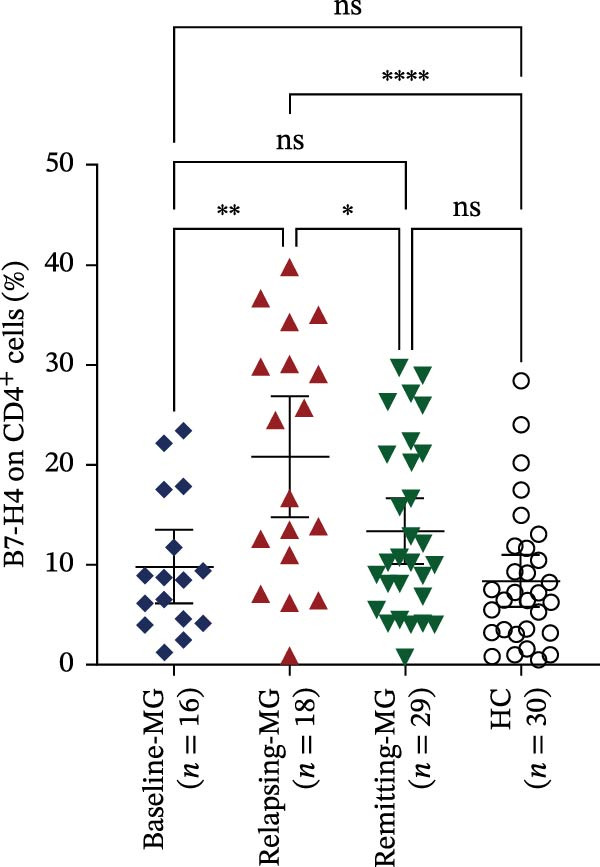
(A)

**Figure:**
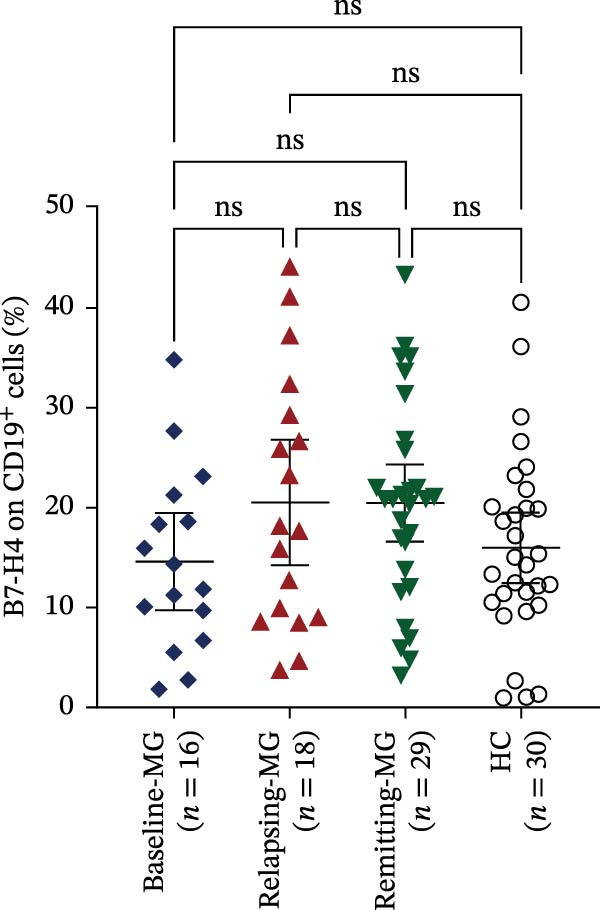
(B)

**Figure:**
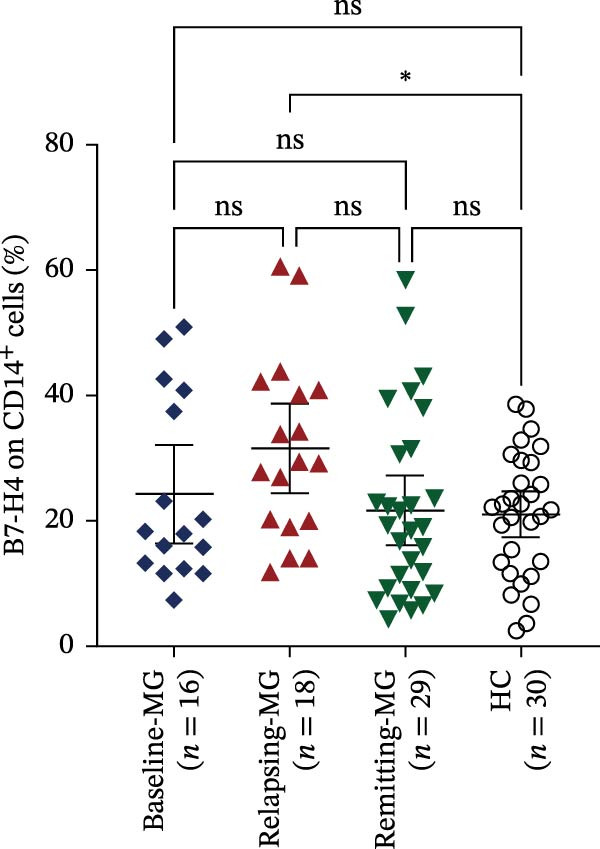
(C)

**Figure:**
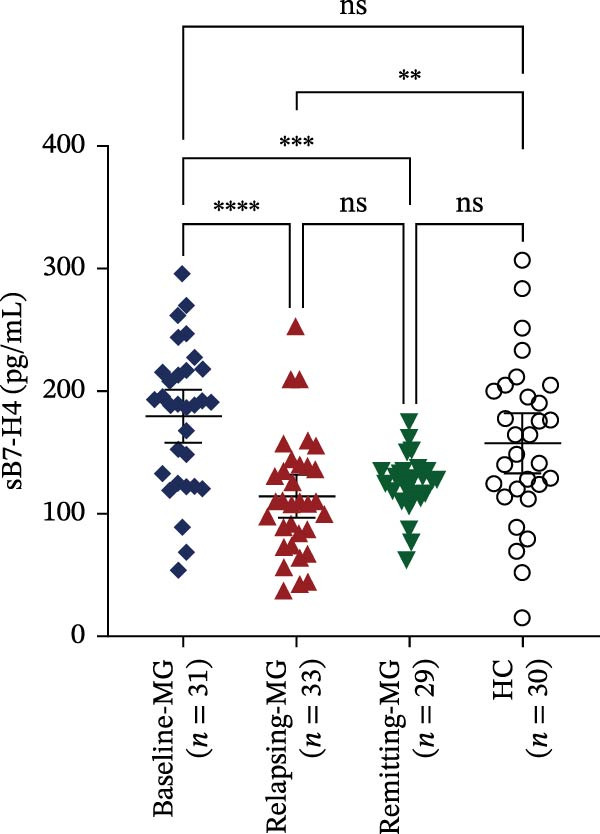
(D)

As shown in Figure 2B, mB7‐H4 expression on CD19^+^ B cells did not differ significantly among HC and MG patients at any disease stage (baseline, relapse, and remission), nor were differences observed among the MG subgroups themselves (all *p* > 0.05).

Regarding CD14^+^ monocytes (Figure 2C), mB7‐H4 expression was significantly upregulated in relapsing‐MG patients compared to HC (*p* < 0.05). However, no significant differences were observed between baseline‐MG or remitting‐MG patients and HC (*p* > 0.05), nor among the three MG subgroups (*p* > 0.05).

Plasma sB7‐H4 was summarized in Figure 2D. Compared with HC, relapsing‐MG patients exhibited significantly reduced sB7‐H4 levels (*p* < 0.05). Notably, sB7‐H4 levels were also significantly lower in both relapsing‐MG and remitting‐MG patients compared to the baseline‐MG group (*p* < 0.05), while no difference was observed between the relapsing and remitting groups (*p* > 0.05).

### 3.3. Stratified Analysis of Plasma sB7‐H4 Levels by Clinical Subgroups

The stratified analysis of plasma sB7‐H4 levels according to clinical characteristics was presented in Table 2. Patients were stratified by sex (male vs. female), age at onset (early vs. late), MGFA classification (OMG vs. generalized MG [GMG]), and thymic pathology (normal vs. abnormal). In the relapsing‐MG group, plasma sB7‐H4 levels were significantly lower in patients with abnormal thymic pathology compared to those with normal thymus (*p* < 0.05). No significant differences were observed across subgroups defined by sex, age at onset, or MGFA classification (*p* > 0.05). In both baseline‐MG and remitting‐MG patients, sB7‐H4 levels did not differ significantly across any of the clinical subgroups examined (all *p* > 0.05).

**Table 2:** Stratified analysis of plasma soluble B7‐H4 levels according to clinical subgroups in patients with myasthenia gravis.

Group	sB7‐H4 (pg/mL)	*t*‐Value	*p*‐Value
Baseline‐MG (*n* = 31)			
Sex			
Male (*n* = 16)	180.76 ± 58.17	0.155	0.878
Female (*n* = 15)	177.44 ± 61.42	—	—
Onset age			
<50 years (*n* = 18)	184.71 ± 52.29	0.613	0.545
≥50 years (*n* = 13)	171.47 ± 68.22	—	—
MGFA classification			
OMG (*n* = 20)	178.67 ± 62.45	0.826	0.951
GMG (*n* = 11)	180.05 ± 54.36	—	—
Thymus pathology			
Normal thymus (*n* = 21)	171.70 ± 64.44	−1.176	0.314
Abnormal thymus (*n* = 10)	194.82 ± 43.45	—	—

Relapsing‐MG (*n* = 33)			

Sex			
Male (*n* = 14)	101.63 ± 43.58	−1.233	0.227
Female (*n* = 19)	122.87 ± 52.43	—	—
Onset age			
<50 years (*n* = 16)	108.47 ± 44.67	−0.603	0.551
≥50 years (*n* = 17)	118.93 ± 54.18	—	—
MGFA classification			
OMG (*n* = 3)	128.37 ± 34.86	0.529	0.601
GMG (*n* = 30)	112.40 ± 50.74	—	—
Thymus pathology			
Normal thymus (*n* = 13)	134.74 ± 53.14	2.059	0.048
Abnormal thymus (*n* = 20)	100.28 ± 42.65	—	—

Remitting‐MG (*n* = 29)			

Sex			
Male (*n* = 9)	121.04 ± 27.34	−0.288	0.775
Female (*n* = 20)	123.73 ± 21.30	—	—
Onset age			
<50 years (*n* = 18)	121.17 ± 20.91	−0.514	0.612
≥50 years (*n* = 11)	125.72 ± 26.58	—	—
MGFA classification			
OMG (*n* = 18)	120.02 ± 22.24	−0.862	0.396
GMG (*n* = 11)	127.60 ± 24.16	—	—
Thymus pathology			
Normal thymus (*n* = 20)	125.91 ± 21.57	1.061	0.298
Abnormal thymus (*n* = 9)	116.19 ± 25.54	—	—

Abbreviations: GMG, generalized myasthenia gravis; MG, myasthenia gravis; MGFA, myasthenia gravis foundation of America; OMG, ocular myasthenia gravis; sB7‐H4, soluble B7‐H4.

### 3.4. Correlation Analysis Between B7‐H4 Levels and Clinical Parameters

To evaluate the clinical relevance of B7‐H4 in MG, correlations between mB7‐H4 (on CD4^+^ T cells, CD19^+^ B cells, and CD14^+^ monocytes) and sB7‐H4 forms with key clinical parameters, including age, QMGs, AchR‐Ab titer, and disease duration, were analyzed (Table 3).

**Table 3:** Correlation analysis between membrane‐bound and soluble B7‐H4 levels and clinical parameters in patients with myasthenia gravis.

Clinical variables	Correlation statistics	B7‐H4 on CD4^+^ cells (%)	B7‐H4 on CD19^+^ cells (%)	B7‐H4 on CD14^+^ cells (%)	sB7‐H4 (pg/mL)
Baseline‐MG					

Age (years)	*r*	0.175	−0.164	0.056	−0.083
	*p*	0.517	0.545	0.837	0.657
	*n*	16	16	16	31
QMGs	*r*	−0.108	0.001	0.330	−0.028
	*p*	0.691	0.996	0.211	0.880
	*n*	16	16	16	31
AchR‐Ab (nmol/L)	*r*	0.302	0.144	0.044	0.077
	*p*	0.255	0.595	0.871	0.682
	*n*	16	16	16	31
Disease duration (months)	*r*	−0.227	−0.410	−0.057	0.061
	*p*	0.417	0.115	0.833	0.743
	*n*	16	16	16	31

Relaping‐MG					

Age (years)	*r*	−0.238	−0.111	−0.105	−0.105
	*p*	0.342	0.661	0.679	0.679
	*n*	18	18	18	33
QMGs	*r*	0.228	0.346	0.544	−0.417
	*p*	0.362	0.160	0.020	0.016
	*n*	18	18	18	33
AchR‐Ab (nmol/L)	*r*	0.042	0.271	0.150	0.188
	*p*	0.868	0.276	0.553	0.295
	*n*	18	18	18	33
Disease duration (months)	*r*	−0.535	−0.333	−0.349	0.138
	*p*	0.022	0.177	0.156	0.444
	*n*	18	18	18	33

Remitting‐MG					

Age (years)	*r*	0.076	−0.358	−0.166	0.051
	*p*	0.694	0.057	0.390	0.791
	*n*	29	29	29	29
QMGs	*r*	0.043	−0.026	−0.220	0.114
	*p*	0.826	0.892	0.252	0.557
	*n*	29	29	29	29
AchR‐Ab (nmol/L)	*r*	0.093	−0.356	−0.193	0.106
	*p*	0.633	0.058	0.316	0.583
	*n*	29	29	29	29
Disease duration (months)	*r*	−0.102	−0.038	−0.251	−0.120
	*p*	0.599	0.845	0.188	0.534
	*n*	29	29	29	29

Abbreviations: AchR‐Ab, acetylcholine receptor antibody; MG, myasthenia gravis; QMGs, quantitative myasthenia gravis score; sB7‐H4, soluble B7‐H4.

In relapsing‐MG patients, mB7‐H4 expression on CD14^+^ monocytes showed a significant positive correlation with QMGs (*r* = 0.544, *p* = 0.020; Figure 3A), whereas sB7‐H4 levels were negatively correlated with QMGs (*r* = −0.417, *p* = 0.016; Figure 3B). In addition, B7‐H4 expression on CD4^+^ T cells was negatively correlated with disease duration (*r* = −0.535, *p* = 0.020). No significant correlations were observed between any form of B7‐H4 and age or AchR‐Ab titer in the relapse cohort.

Figure 3Correlation of B7‐H4 expression with disease severity and therapeutic changes in myasthenia gravis (MG) patients. (A) Correlation between the percentage of B7‐H4 on CD14^+^ monocytes and quantitative myasthenia gravis scores (QMGs) in relapsing‐MG patients (*n* = 18). (B) Correlation between plasma soluble B7‐H4 (sB7‐H4) levels and QMGs in relapsing‐MG patients (*n* = 33). (C) QMGs in MG patients (*n* = 13) at acute exacerbation and after convalescence following immunosuppressive therapy. (D) Plasma sB7‐H4 levels in MG patients (*n* = 13) during acute exacerbation and after convalescence following immunosuppressive therapy. Data in (A) and (B) were analyzed by Pearson’s correlation coefficient (*r* and *p* values as indicated). Statistical significance in (C) and (D) was determined by paired *t*‐test for comparisons between time points within each subgroup.  ^∗∗∗^ *p* < 0.001; ns, not significant.

**Figure:**
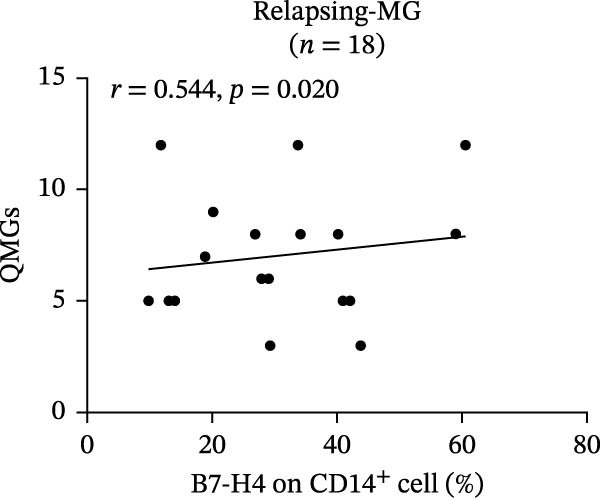
(A)

**Figure:**
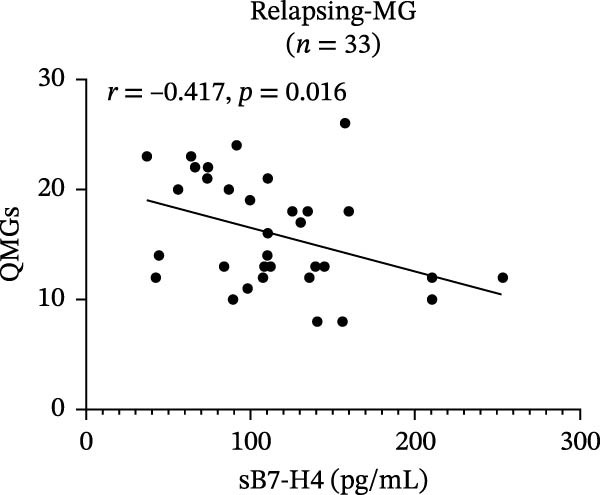
(B)

**Figure:**
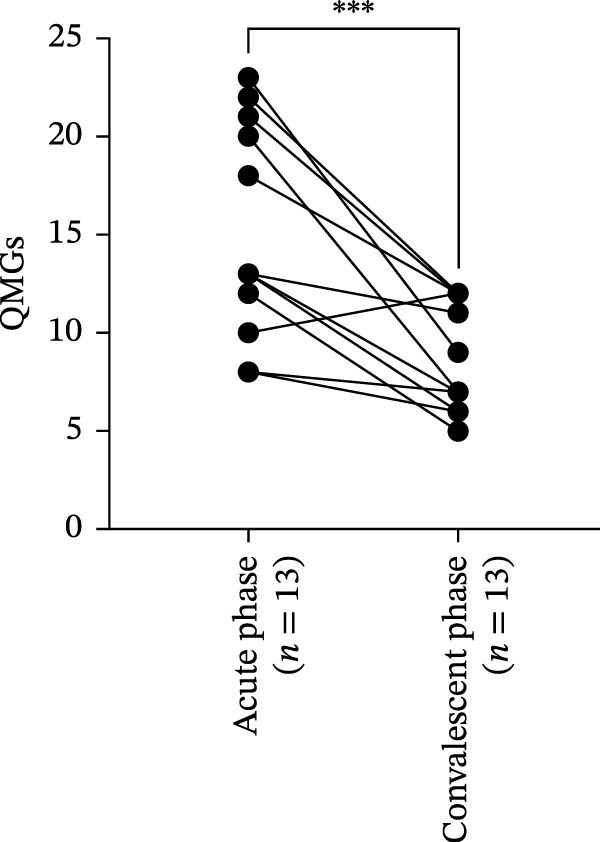
(C)

**Figure:**
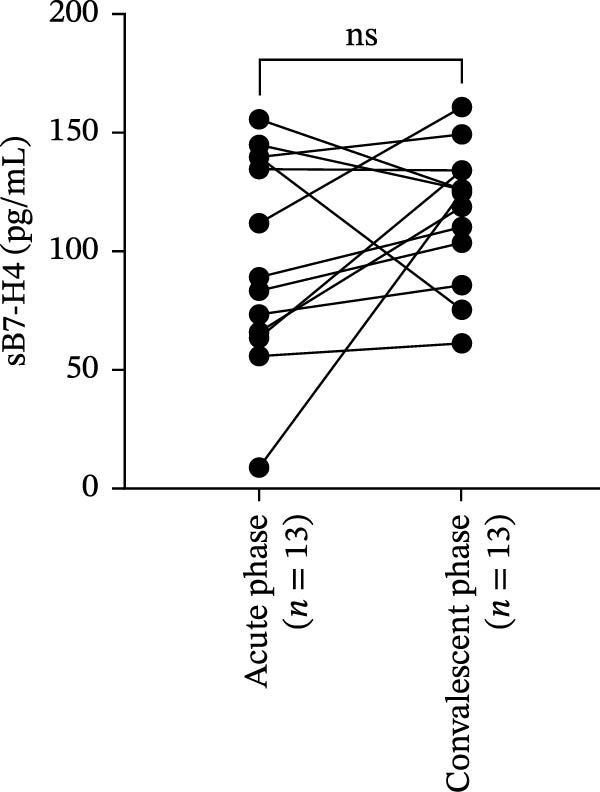
(D)

In contrast, neither membrane‐bound nor sB7‐H4 levels showed any significant correlation with the clinical parameters evaluated in patients with baseline‐MG or remitting‐MG (all *p* > 0.05).

### 3.5. Changes in Plasma sB7‐H4 Levels Before and After Immunosuppressive Therapy in MG Patients

To assess the impact of immunosuppressive therapy on sB7‐H4 expression, paired plasma samples from 13 MG patients were retrospectively analyzed during acute exacerbation and following clinical convalescence. All patients received intravenous methylprednisolone (1–1.5 mg/kg/day) as the core immunosuppressive regimen during the acute phase, with three patients additionally receiving sequential tacrolimus (0.05–0.1 mg/kg/day).

As expected, QMGs were significantly reduced in the convalescent phase compared to the acute exacerbation phase (*p* < 0.05; Figure 3C), confirming the clinical improvement after the treatment. However, plasma sB7‐H4 levels did not differ significantly before and after immunosuppressive therapy (*p* > 0.05; Figure 3D). These results suggest that sB7‐H4 expression may be not modulated by short‐term immunosuppressive treatment, even in the context of effective clinical response.

### 3.6. Logistic Regression Analysis of Relapse Risk

Univariate and multivariate logistic regression analyses evaluating risk factors for MG relapse in the membrane B7‐H4 cohort were presented in Table 4. In univariate analysis, immunosuppressant use emerged as a significant protective factor (OR < 1, *p* < 0.05), whereas high mB7‐H4 expression on CD4^+^ T cells, GMG, thymic abnormality, and elevated AchR‐Ab titers were significant risk factors (OR > 1, *p* < 0.05). After adjustment for potential confounders, only MGFA classification (adjusted OR = 22.019, 95% CI: 2.162–224.260, *p* = 0.009) and immunosuppressant treatment (adjusted OR = 0.076, 95% CI: 0.007–0.785, *p* = 0.031) remained independently associated with relapse. These results indicate that patients with GMG had an approximately 21‐fold higher risk of relapse than those with ocular MG, while combination immunosuppressant therapy reduced relapse risk by approximately 92.4% compared to glucocorticoid monotherapy.

**Table 4:** Univariate and multivariate logistic regression analysis of relapse risk in the membrane‐bound B7‐H4 cohort.

Variables	Univariate analysis				Multivariate analysis			
	B	SE	*p*	OR (95% CI)	B	SE	*p*	Adjusted OR (95% CI)
CD4^+^B7‐H4 (%)	0.070	0.031	0.025	1.072 (1.009–1.131)	0.046	0.043	0.290	1.047 (0.962–1.140)
CD19^+^B7‐H4 (%)	0.000	0.028	0.987	1.000 (0.948–1.056)	—	—	—	—
CD14^+^B7‐H4 (%)	0.042	0.021	0.051	1.043 (1.000–1.087)	—	—	—	—
Sex, *n* (%)								
Male	Ref	—	—	—	—	—	—	—
Female	0.388	0.698	0.578	1.474 (0.375–5.790)	—	—	—	—
Age (years)	0.002	0.017	0.907	1.002 (0.970–1.035)	—	—	—	—
Disease duration (months)	−0.006	0.006	0.315	0.994 (0.982–1.006)	—	—	—	—
MGFA classification								
OMG	Ref	—	—	—	—	—	—	—
GMG	2.575	0.757	0.001	13.125 (2.978–57.839)	3.091	1.184	0.009	22.019 (2.162–224.206)
Thymus pathology								
Normal thymus	Ref	—	—	—	—	—	—	—
Abnormal thymus	1.344	0.658	0.041	3.833 (1.057–13.909)	0.887	1.061	0.403	2.427 (0.303–19.433)
AchR‐Ab (nmol/L)	0.029	0.079	0.000	1.377 (1.145–1.562)	0.157	0.129	0.224	1.170 (0.908–1.507)
Treatment								
Glucocorticoid	Ref	—	—	—	—	—	—	—
Immunosuppressant	−1.250	0.628	0.047	0.286 (0.084–0.981)	−2.581	1.193	0.031	0.076 (0.007–0.785)

Abbreviations: AchR‐Ab, acetylcholine receptor antibodies; B, beta coefficient; CI, confidence interval; GMG, generalized myasthenia gravis; MGFA, myasthenia gravis foundation of America; OMG, ocular myasthenia gravis; OR, odds ratio; SE, standard error.

In the sB7‐H4 cohort, univariate logistic regression identified GMG, thymic abnormalities, elevated AChR‐Ab titers, and glucocorticoid monotherapy as significant factors associated with MG relapse (all *p*  < 0.05; Table 5). Multivariable regression analysis demonstrated that GMG (vs. OMG; adjusted OR = 5.543, 95% CI: 1.023–30.038, *p* = 0.047), elevated AChR‐Ab titers (per unit increase; adjusted OR = 1.241, 95% CI: 1.022–1.507, *p* = 0.029), and combination therapy with immunosuppressants (vs. glucocorticoid monotherapy; adjusted OR = 0.186, 95% CI: 0.045–0.768, *p* = 0.020) was independently associated with relapse risk. Specifically, relapse risk was 5.543‐fold higher in GMG than OMG patients, increased by 24.1% per unit rise in AChR‐Ab titers, and was reduced by 81.4% with combination immunosuppressants versus glucocorticoid monotherapy.

**Table 5:** Univariate and multivariate logistic regression analysis of relapse risk in the soluble B7‐H4 cohort.

Variables	Univariate analysis				Multivariate analysis			
	*B*	SE	*p*	OR (95% CI)	*B*	SE	*p*	Adjusted OR (95% CI)
sB7‐H4 (pg/mL)	−0.006	0.007	0.366	0.994 (0.981–1.007)	—	—	—	—
Sex, *n* (%)								
Male	Ref	—	—	—	—	—	—	—
Female	−0.493	0.534	0.356	0.611 (0.214–1.739)	—	—	—	—
Age (years)	0.020	0.017	0.240	1.020 (0.987–1.056)	—	—	—	—
Disease duration (months)	−0.001	0.004	0.751	0.999 (0.990–1.007)	—	—	—	—
MGFA classification								
OMG	Ref	—	—	—	—	—	—	—
GMG	2.795	0.716	0.000	16.364 (4.019–66.623)	1.713	0.862	0.047	5.543 (1.023–30.038)
Thymus pathology								
Normal thymus	Ref	—	—	—	—	—	—	—
Abnormal thymus	1.229	0.537	0.022	3.419 (1.194–9.788)	0.623	0.718	0.386	1.864 (0.456–7.623)
AchR‐Ab (nmol/L)	0.290	0.079	0.000	1.337 (1.145–1.562)	0.216	0.099	0.029	1.241 (1.022–1.507)
Treatment								
Glucocorticoid	Ref	—	—	—	—	—	—	—
Immunosuppressant	−1.381	0.565	0.015	0.251 (0.083–0.761)	−1.680	0.722	0.020	0.186 (0.045–0.768)

Abbreviations: AchR‐Ab, acetylcholine receptor antibodies; B, beta coefficient; CI, confidence interval; GMG, generalized myasthenia gravis; MGFA, myasthenia gravis foundation of America; OMG, ocular myasthenia gravis; OR: odds ratio; SE, standard error; sB7‐H4, soluble B7‐H4.

## 4. Discussion

The B7 family of costimulatory molecules is essential for regulating T cell responses [5]. Among them, B7‐H4 is an immune checkpoint ligand that has emerged as a promising molecular target, particularly due to its expression in a wide range of solid tumors [16]. Increasing evidence has also implicated B7‐H4 in the pathogenesis of autoimmune diseases, including multiple sclerosis [25], type 1 diabetes [29], RA [30], and SLE [20]. However, the expression patterns and immunological functions of B7‐H4 in MG have remained largely unexplored. In this study, we systematically examined both mB7‐H4 on immune cells and sB7‐H4 in plasma across different disease stages in MG, and evaluated their clinical relevance.

To our knowledge, this is the first study to characterize B7‐H4 expression in the peripheral blood of MG patients at distinct disease phases. We demonstrate that mB7‐H4 expression is significantly upregulated on CD4^+^ T cells and CD14^+^ monocytes specifically during disease relapse. This phase‐specific increase suggests that B7‐H4 expression is dynamically regulated rather than constitutively elevated, and may actively participate in periods of heightened autoimmune activity. B7‐H4, a recently identified member of the B7 family, is known to be expressed activated T cells, B cells, monocytes, and DCs [31], and functions predominantly as a negative regulator of T‐cell immunity by inhibiting T‐cell proliferation, cytokine production, and cell‐cycle progression [6]. MG relapse, which is characterized by breakdown of immune tolerance and amplified T‐cell‐mediated pathology, may therefore represent a strong trigger for the induction of this inhibitory pathway. We propose that during relapse, upregulation of B7‐H4 on CD4^+^ T cells functions as an intrinsic compensatory mechanism to restrain pathogenic T‐cell expansion and effector function, while its increased expression on monocytes enables a paracrine immunosuppressive interaction between innate and adaptive immune compartments.

In addition to its membrane‐bound form, B7‐H4 also exists as a soluble protein in circulation [32, 33]. Soluble costimulatory molecules are generally generated through proteolytic cleavage or alternative mRNA splicing of membrane‐bound counterparts [27]. In contrast to the upregulation of mB7‐H4, we observed a significant reduction in plasma sB7‐H4 levels in patients with relapsing MG compared with HCs. The mechanisms underlying this discordant regulation remain unclear. While sB7‐H4 is elevated in several malignancies [32, 34, 35], its reduction in relapsing MG suggests a distinct immunoregulatory role in autoimmune settings. Experimental studies have shown that sB7‐H4 can function as a decoy molecule, competitively inhibiting the interaction between mB7‐H4 and its receptor, thereby enhancing T‐cell‐mediated autoimmune responses [36]. Accordingly, the reduction in sB7‐H4 levels during MG relapse may enhance mB7‐H4/B7‐H4R interactions, thus inhibiting abnormal T‐cell proliferation and activation. Taken together, our findings may support a model in which inflammatory activation during MG relapse engages the B7‐H4 pathway, characterized by coordinated upregulation of mB7‐H4 and downregulation of sB7‐H4, synergistically dampening immune activation and limiting excessive autoimmune damage.

Compared with baseline MG, plasma sB7‐H4 levels were significantly reduced in both relapsing and remitting patients. Although not statistically significant, sB7‐H4 levels tended to be higher during remission than during relapse. We speculate that these fluctuations reflect dynamic reprogramming of B7‐H4 regulation across disease stages. Elevated sB7‐H4 levels at baseline may contribute to a state of systemic immune homeostasis. During relapse, the pro‐inflammatory microenvironment may suppress proteolytic shedding of sB7‐H4, shifting immune regulation toward localized, membrane‐bound, contact‐dependent inhibition. As inflammation resolves and remission ensues, partial restoration of proteolytic activity may lead to a rebound in sB7‐H4 levels. However, incomplete immune reconstitution may prevent full normalization.

To further explore the clinical relevance of B7‐H4, we analyzed its association with clinical features across disease phases. In relapsing MG patients, sB7‐H4 levels were significantly lower in those with abnormal thymic pathology than in patients with a normal thymus. Although B7‐H4 overexpression has been extensively reported in several malignancies, including lung cancer [37], breast cancer [38], endometrial and ovarian tumors [16, 39], and its role in thymoma remains poorly characterized. We hypothesize that thymoma may evade immune surveillance by downregulating sB7‐H4, thereby enhancing mB7‐H4‐mediated suppression of T‐cell function [40]. Furthermore, mB7‐H4 expression on CD4^+^ T cells was negatively correlated with disease duration in relapsing MG, suggesting that the capacity to mount this compensatory inhibitory response may diminish with prolonged disease. Consistent with findings in SLE [21], we observed a negative correlation between sB7‐H4 levels and disease severity, as assessed by QMGs. Conversely, mB7‐H4 expression on CD14^+^ monocytes was positively correlated with QMGs, indicating that B7‐H4 expression reflects disease severity in a context‐dependent manner. Given the limited sample size, these associations should be interpreted cautiously.

Previous studies have suggested that glucocorticoids can modulate sB7‐H4 expression [41, 42]. In our paired analysis, plasma sB7‐H4 levels tended to increase following immunosuppressive therapy, although the difference did not reach statistical significance. These findings suggest that immunosuppressants may exert a modest influence on sB7‐H4 expression. However, larger longitudinal studies are required to determine the robustness and clinical relevance of this effect.

Logistic regression analyses further clarified the role of B7‐H4 in MG relapse. In the mB7‐H4 cohort, although high CD4^+^ T‐cell B7‐H4 expression was associated with relapse in univariate analysis, it did not remain an independent predictor after adjustment for confounders. This finding indicates that B7‐H4 upregulation reflects underlying disease activity rather than serving as an autonomous driver of relapse. In contrast, analysis of the sB7‐H4 cohort identified GMG, elevated AChR antibody titers, and glucocorticoid monotherapy as independent predictors of relapse. Importantly, both cohorts consistently identified GMG as a strong risk factor for relapse and combination immunosuppressive therapy as protective. These results support a clear clinical implication: patients with GMG represent a high‐risk subgroup that may benefit from early and aggressive combination immunosuppressive strategies to prevent disease recurrence. This study has several strengths. First, it provides the first comprehensive evaluation of both membrane‐bound and sB7‐H4 dynamics in AChR‐Ab‐positive MG across disease stages. Second, it identifies a relapse‐associated activation pattern of the B7‐H4 pathway, characterized by coordinated regulation of its membrane and soluble forms. Third, it reveals distinct and opposing correlations between B7‐H4 forms and disease severity. Fourth, it clarifies the role of CD4^+^ T‐cell B7‐H4 as a disease activity–associated marker rather than an independent relapse predictor. Finally, it reinforces the clinical importance of early immunosuppressive intervention, particularly in patients with generalized disease.

Several limitations should be acknowledged. The single‐center design and modest sample size may limit generalizability. The cross‐sectional nature of the study and the use of nonoverlapping cohorts for mB7‐H4 and sB7‐H4 analyses preclude causal inference. Moreover, mechanistic insights into B7‐H4 signaling in MG remain indirect. Future studies should incorporate large‐scale longitudinal cohorts and mechanistic investigations using experimental autoimmune MG models and in vitro systems to definitively determine whether B7‐H4 signaling exerts a protective role in MG.

## 5. Conclusions

In summary, we demonstrate that MG relapse is characterized by upregulation of mB7‐H4 and concomitant downregulation of sB7‐H4, with both forms correlating with disease severity. Thymic abnormalities may contribute to reduced sB7‐H4 levels, while immunosuppressive therapy may have limited short‐term effects on its expression. Although CD4^+^ T‐cell B7‐H4 expression is associated with relapse, it is not an independent predictor. Collectively, our findings suggest that inflammatory activation during MG relapse engages the B7‐H4 pathway, wherein increased mB7‐H4 and decreased sB7‐H4 act in concert to suppress immune activation, functioning as a compensatory brake to limit autoimmune damage.

**Nomenclature**
MG: Myasthenia gravis
HC: Healthy control
PB: Peripheral blood
mB7‐H4: Membrane B7‐H4
sB7‐H4: Soluble B7‐H4
ELISA: Enzyme‐linked immunosorbent assay
OMG: Ocular myasthenia gravis
GMG: Generalized myasthenia gravis
QMGs: Quantitative myasthenia gravis scores
AchR‐Ab: Acetylcholine receptor antibodies
MHC: Major histocompatibility complex
SLE: Systemic lupus erythematosus
RA: Rheumatoid arthritis
MGFA: Myasthenia Gravis Foundation of America
PIS: Postintervention status
MMF: Mycophenolate Mofetil
RTX: Rituximab
CTX: Cyclophosphamide.

## Author Contributions

Conceptualization: Xiaoling Zhou and Qun Xue. Methodology: Xiaoling Zhou and Yunfei Zhu. Software: Jingluan Tian and Xiaoling Zhou. Validation, supervision, funding acquisition: Qun Xue. Formal analysis, writing – original draft preparation, visualization: Xiaoling Zhou. Investigation: Yunfei Zhu and Tiantian Gui. Resources: Yanzheng Gu and Haifeng Lu. Data curation: Xiaoling Zhou and Wentong Ju. Writing – review and editing: Qun Xue and Jingluan Tian.

## Funding

This study was supported by grants from the National Natural Science Foundation of China (Grant 82371365), the Soochow University Collaborative Innovation Center Research Project (Grants H230028, H240669, H240216, H231180, and H240827), and The First Affiliated Hospital of Soochow University Clinical Diagnosis and Treatment Technology Innovation Program (Grant 0302020301030).

## Disclosure

All authors have read and agreed to the published version of the manuscript. All AI‐generated content has been reviewed and approved by the authors, who are solely responsible for the final manuscript.

## Conflicts of Interest

The authors declare no conflicts of interest.

## Supporting Information

Additional supporting information can be found online in the Supporting Information section.

## Supporting information


**Supporting Information** Figure S1. It presents the gating strategy for evaluating B7‐H4 expression on CD4^+^ T cells, CD19^+^ B cells, and CD14^+^ monocytes. For each cell population, the region was first defined based on forward and side scatter characteristics, followed by identification using specific surface markers (CD4, CD19, or CD14). The gate for B7‐H4 positivity was set according to fluorescence minus one (FMO) controls, ensuring accurate discrimination of specific staining.

## Data Availability

The authorship will provide all raw data supporting the conclusions of this manuscript without any reservation.
