# Supplementary material for: Clinical Significance of B7‐H4 in Peripheral Blood of Patients With Myasthenia Gravis
Source: J Immunol Res. 2026 May 9;2026:2743928. doi: 10.1155/jimr/2743928 (PMC13157312; doi:10.1155/jimr/2743928)
Supplement: Supplementary file 1 — Supporting Information Figure S1. It presents the gating strategy for evaluating B7‐H4 expression on CD4+ T cells, CD19+ B cells, and CD14+ monocytes. For each cell population, the region was first defined based on forward and side scatter characteristics, followed by identification using specific surface markers (CD4, CD19, or CD14). The gate for B7‐H4 positivity was set according to fluorescence minus one (FMO) controls, ensuring accurate discrimination of specific staining. [file JIMR-2026-2743928-s001.docx]

**Supplementary Materials**

Xiaoling Zhou^1,2^^†^, Yunfei Zhu^3†^, Tiantian Gui^4^, Yanzheng Gu^2^, Haifeng Lu^1^, Wentong Ju^1^, Jingluan Tian^1,2^*****, Qun Xue^1,2^******

1. Department of Neurology, The First Affiliated Hospital of Soochow University, Suzhou, Jiangsu, 215000, China.
2. Institute of Clinical Immunology, Jiangsu Key Laboratory of Clinical Immunology, The First Affiliated Hospital of Soochow University, Suzhou, Jiangsu, 215006, China.
3. Department of Neurology, The Fourth Affiliated Hospital of Soochow University, Suzhou, Jiangsu, 215123, China.
4. Department of Neurology, The People's Hospital of Guangxi Zhuang Autonomous Region, Guangxi Academy of Medical Sciences, Nanning, Guangxi, 530021, China.

†These authors contributed equally to this work.

**** Correspondence:**

Qun Xue

qxue_sz@163.com

*** Correspondence:**

Jingluan Tian

jingluan@163.com


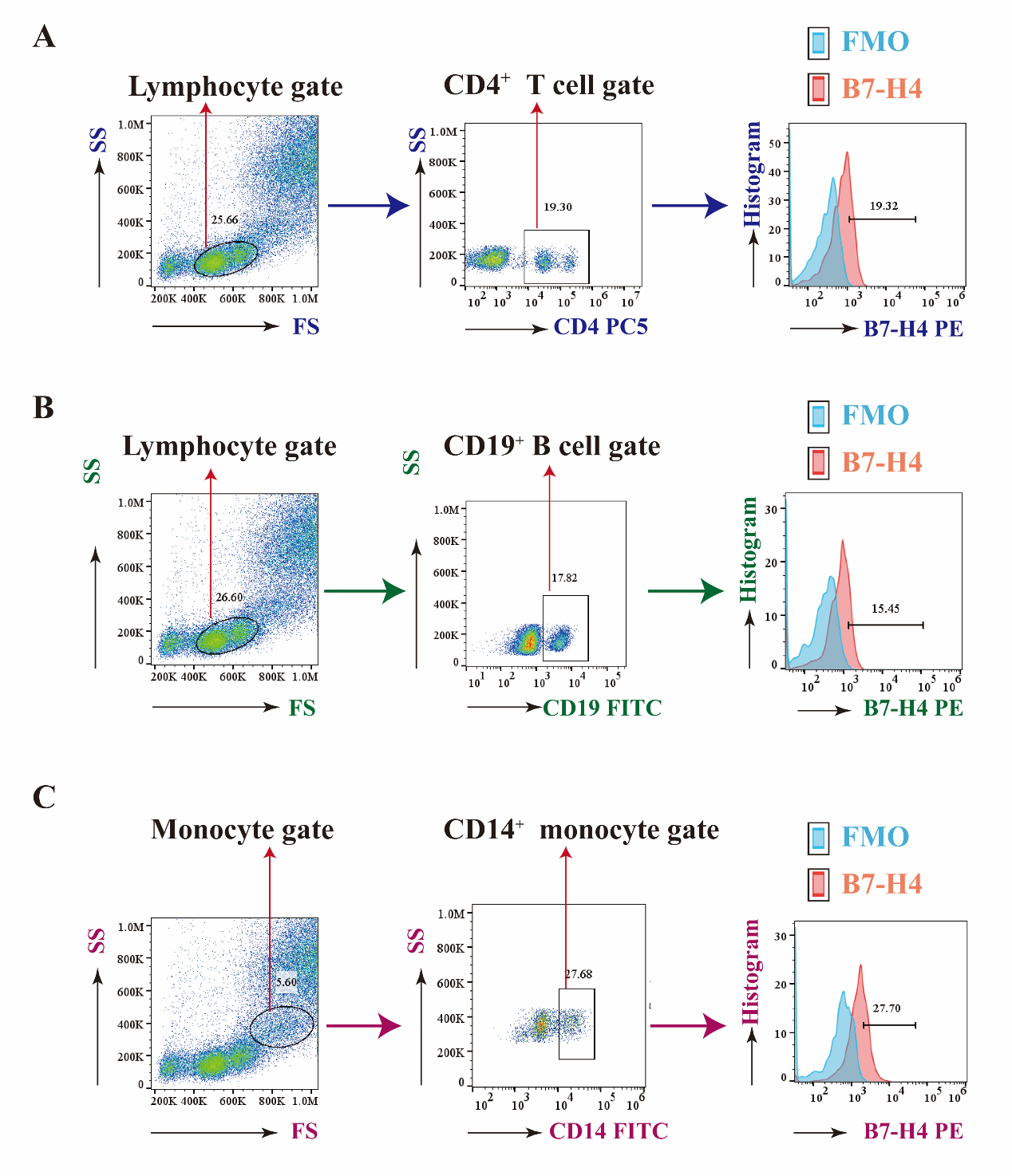


**Figure S1. Gating strategy for B7H4 expression on CD4⁺ T cells, CD19⁺ B cells, and CD14⁺ monocytes.**

**(A) Gating strategy for B7H4 expression on CD4⁺ T cells.** Lymphocytes were first gated on an FSC-A vs SSC-A dot plot (left panel, lymphocyte gate). From the lymphocyte gate, CD4⁺ T cells were identified based on CD4-PC5 expression (middle panel, CD4⁺ T cell gate). The right panel shows a histogram overlay of B7H4-PE expression on gated CD4⁺ T cells. The blue histogram represents the fluorescence minus one (FMO) control (Tube ①: CD4-PC5 only), and the red histogram represents the fully stained sample (Tube ②: CD4-PC5 + B7H4-PE). The gate for B7H4⁺ cells was set based on the FMO control.

**(B) Gating strategy for B7H4 expression on CD19⁺ B cells.** Lymphocytes were gated on FSC-A vs SSC-A (left panel), followed by gating of CD19⁺ B cells based on CD19-FITC expression (middle panel). The right panel shows B7H4-PE expression on gated CD19⁺ B cells. The blue histogram represents the FMO control (Tube ③: CD19-FITC only), and the red histogram represents the fully stained sample (Tube ④: CD19-FITC + B7H4-PE). The gate for B7H4⁺ cells was set based on the FMO control.

**(C) Gating strategy for B7H4 expression on CD14⁺ monocytes.** Monocytes were first gated on FSC-A vs SSC-A based on their characteristic size and granularity (left panel, monocyte gate). From the monocyte gate, CD14⁺ monocytes were identified based on CD14-FITC expression (middle panel, CD14⁺ monocyte gate). The right panel shows B7H4-PE expression on gated CD14⁺ monocytes. The blue histogram represents the FMO control (Tube ⑤: CD14-FITC only), and the red histogram represents the fully stained sample (Tube ⑥: CD14-FITC + B7H4-PE). The gate for B7H4⁺ cells was set based on the FMO control.
